# The synthesis of controlled shape nanoplasmonic silver-silica structures by combining sol-gel technique and direct silver reduction

**DOI:** 10.1186/s11671-015-0839-x

**Published:** 2015-03-19

**Authors:** Lina Ramanauskaite, Valentinas Snitka

**Affiliations:** Research Center for Microsystems and Nanotechnology, Kaunas University of Technology, Studentu 65, LT-51369 Kaunas, Lithuania

**Keywords:** Sol-gel, Silver nanoparticles, SERS, PEG 400, Nanoplasmonic silver structures

## Abstract

In this work, we have obtained nanoplasmonic silver structures deposited on the glass substrates by combining sol-gel technology and direct silver ion reduction on the film surfaces. The key point of the work was the usage of polyethylene glycol 400 (PEG 400) both as the pore former and reducing agent for silver ions. We have investigated the influence of PEG 400 amount on the formation of silver nanoparticles on the film surface. It was found that control of PEG 400 amount in the sols allows the creation of porous films with specific organized silver nanoparticles or clusters on the surface. Optical, morphological and structural characteristics of the structures were measured and studied. Atomic force microscopy (AFM) and scanning electron microscopy (SEM) were used for nanostructure size and shape characterization. We were able to form a 40- to 200-nm-diameter ring-type, spherical and self-assembled nanoparticles on the film surface. The results of UV-vis absorbance spectra have shown the high quality of plasmonic structures with plasmon resonance wavelength in the region between 470 and 480 nm. The synthesized silica films decorated with silver nanoparticles were tested as substrates for the surface-enhanced Raman spectroscopy (SERS) and showed an enhancement relative to micro-Raman of more than 200 times.

## Background

Since their discovery in 1957, surface plasmons have been exploited in applications as diverse as single-molecule surface-enhanced Raman spectroscopy (SERS), nanoscale optical modulators, high-efficiency solar cells, nanoscale lasers, biological rulers and electromagnetic meta-materials for invisibility and sub-diffraction-limited optical microscopy [1]. Noble metal nanoparticles supporting plasmonic resonances behave as efficient nanosources of light, heat and energetic electrons. Owing to these properties, they offer a unique playground to trigger chemical reactions at the nanoscale [2]. Therefore, the emergence of the field of plasmonics - the science and engineering of electromagnetic field interactions with metallic nanostructures - has a broad scope. However, the precise understanding of the various types of plasmon resonances has a great interest in development and application of plasmonic substrates. Most of the plasmonic substrates are 2D planar systems which limit the active area to a single Cartesian plane. The fabrication of 3D plasmonic substrates with the aim to extend the SERS hot spots into the third dimension along the *z*-axis is a way to open new applications of plasmonic structures. There are various methods such as lithography [3-5], laser ablation [6,7], sputter coating [8] or chemical synthesis [9,10] used for the preparation of plasmonic structures. All these methods allow obtaining nanostructures of desirable sizes, shapes or arrangements which are very important for high-sensitivity Raman measurements. One of the most important parameters determining the sensitivity of SERS substrates is the shape of nanoparticles. For example, sharp-edged silver nanotriangles or nanocubes produce high electromagnetic field at the edges of the nanoparticles and result in a strongly enhanced Raman signal [11,12]. Another example is a dimer-type plasmonic nanostructure separated by the nanogaps. Electromagnetic field generated in the nanosized gaps is one of the main enhancement mechanisms to realize a single-molecule SERS [13].

The chemical preparation of SERS substrates often includes classical sol-gel technology [14-16] attractable for most authors because of its simplicity and inexpensiveness. This method allows the formation of the films on various surfaces directly from the solution, control the porosity by varying synthesis parameters or using organic templates, functionalization of the films with noble metals nanoparticles as well as the formation of self-assembled structures. In this paper, we propose a new chemical route for the synthesis of hybrid silica films using polyethylene glycol 400 (PEG 400) as porogen and reducing agent. Within a few years, a number of publications represented polyethylene glycol as a perfect substance for “green reduction” of silver ions and stabilization of silver nanoparticles [17-21]. It also became attractive to its solubility in aqueous media, low toxicity and wide selection of molecular weights [22]. In our work, we present a novel methodology for the preparation of 3D silica films decorated with silver nanostructures by combining sol-gel technology and direct silver ion reduction and demonstrate the possibility to control the shape of synthesized plasmonic structures from the ring up to the networked nanoparticles. The efficiency of the fabricated SERS substrates for the enhancement of Raman signal was tested using crystal violet dye.

## Methods

### Materials

For the synthesis of hybrid silica films, ethanol, water, hydrochloric acid, PEG 400 and silver nitrate were purchased from Sigma-Aldrich (St. Louis, MO, USA) and tetraethylortosilicate (TEOS) were from Acros Organics (Geel, Belgium). All the reagents were analytical grades and used without further purification.

### Preparation of silver nanoparticle-decorated silica films

**Figure 1:**
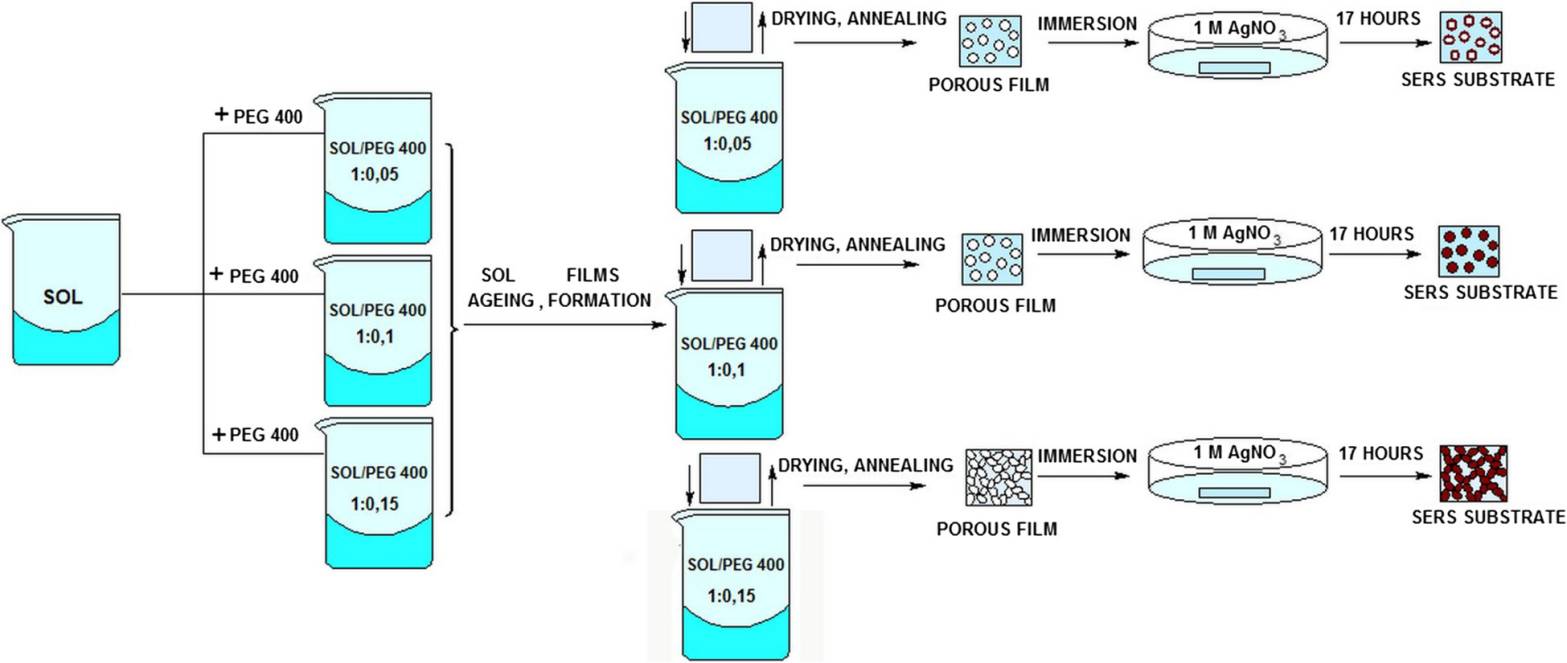
**Illustration of the synthesis of the hybrid silica films.**

The sol was prepared by mixing TEOS, ethanol and water in the flask with ratios 0.2:0.4:1, respectively. Reaction was carried out under the acidic conditions: hydrochloric acid was used to reach the pH value of 2.3. The solution was mixed for 1 h maintaining a constant temperature of 60°C and finally divided into three flasks. Pure PEG 400 was added to reach sol/PEG ratios of 1:0.05 (*v*:*v*), 1:0.10 (*v*:*v*) and 1:0.15 (*v*:*v*) in each flask, respectively. All PEG 400-modified sols were aged for 48 h at room temperature. The films were formed on the ethanol-cleaned microscopic glasses by spin-coating method with a spin speed of 1,500 rpm and a spin time of 10 s, dried at room temperature and heated in air at 300°C for 2 h. After the thermal treatment, all the films were cooled to room temperature, immersed into freshly prepared 1 M AgNO_3_ solution for 17 h, then removed from the solution and dried under the nitrogen flow. Schematic illustration of the synthesis is represented in Figure 1.

### Characterization

Optical characterization of silver nanoparticle-modified silica films was carried out using UV-vis USB 2000 optic spectrometer (Ocean-Optics Inc., Dunedin, FL, USA). Atomic force microscope images were obtained on scanning probe microscope (NT-MDT Inc., Moscow, Russia) in a semi-contact tapping mode using commercial cantilevers (Tap 150 Al-G, Budget Sensors, Sofia, Bulgaria). Scanning electron microscope (Helios NanoLab 650, FEI, Holland, The Netherlands) was used for the structural investigation of the silver nanoparticle-decorated silica films. Raman and SERS measurements were performed on Raman spectrometer (NTEGRA Spectra, NT-MDT, Moscow, Russia) in inverted mode using green laser (532 nm) as excitation source and 30-s acquisition time at laser power of 5 mW.

## Results and discussion

**Figure 2:**
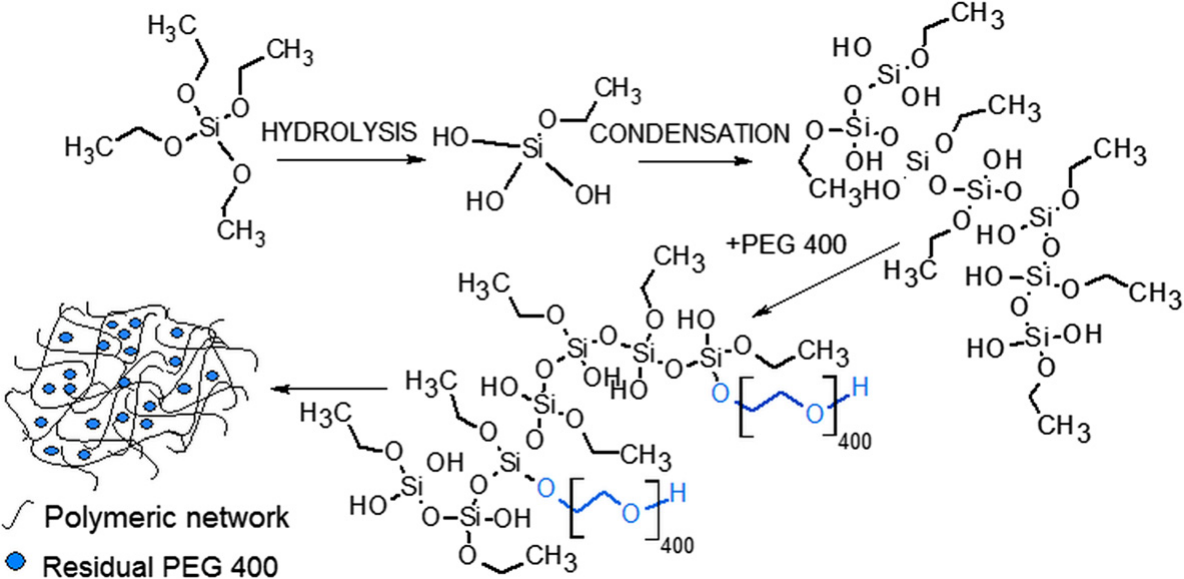
**Proposed mechanism of PEG 400 interaction with silane network.**

Sol-gel technology is a process involving hydrolysis, water and alcohol condensation. In a general case, hydrolysis is based on the replacement of alkoxide ligand by a hydroxyl group. During the condensation stage occurring between two silanols or silanol and alkoxide, molecular weight of the synthesis product is increasing. When PEG 400 is introduced into the reaction system, it chemically interacts with sol particles thus resulting in the formation of hybrid polymeric network. Chemical interplay takes place via hydrogen bonding between oxygen atoms of PEG and silanol groups [23], whereas part of PEG 400 interacts physically with sol particles and remains inside the pore channels [24] (Figure 2).

**Figure 3:**
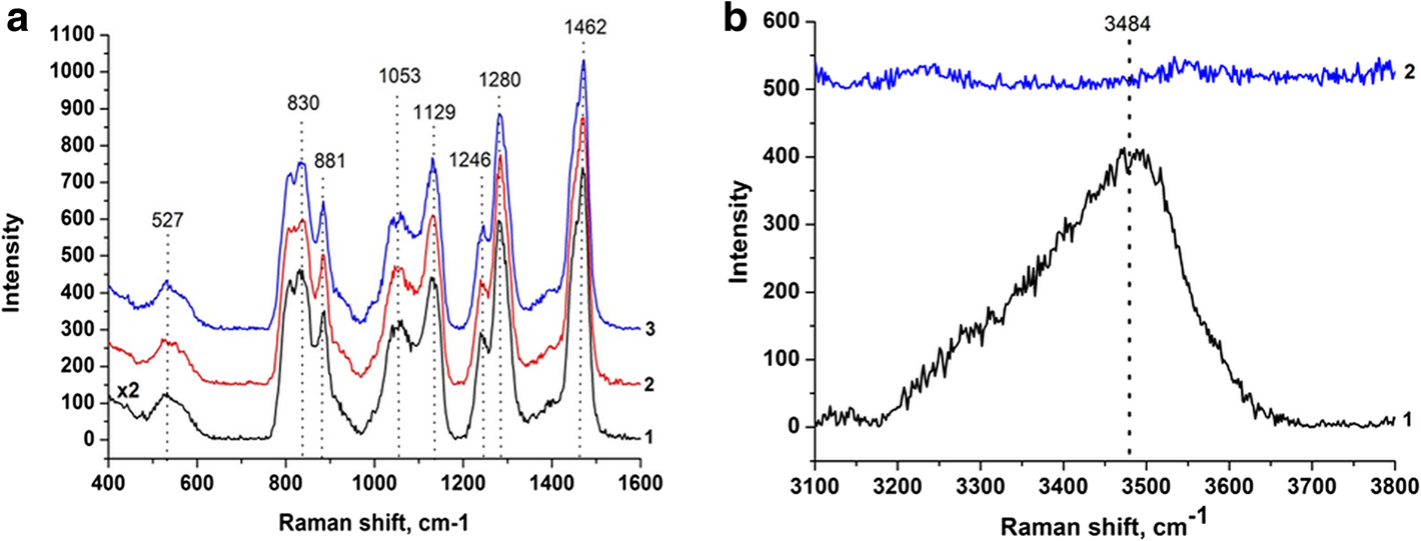
**Raman spectrum of PEG 400-modified silica films after thermal treatment at 300°C. (a)** A film prepared at a volume ratio of sol:PEG 1:0.05 (1); a film prepared at a volume ratio of sol:PEG 1:0.10 (2); a film prepared at a volume ratio of sol:PEG 1:0.15 (3); **(b)** a film prepared at a volume ratio of sol:PEG 1:0.10 before the deposition of silver nanoparticles showing a band of -OH vibrations responsible for the reduction of silver ions (1) and after the deposition of silver nanoparticles showing the absence of these vibrations and suggesting the complete reduction reaction (2).

During the thermal annealing of hybrid silica films, chemically unreacted PEG 400 is not fully removed from the polymeric matrix. This is confirmed by Raman measurements performed after thermal treatment of the films (Figure 3).

Raman spectra of organically modified silicates strongly depend on hybrid material preparation technique, molecular weight, concentration and nature of modifying polymer. Considering to this, in Figure 3a, the peaks at approximately 830 and 881 cm^−1^ as well as 1,053 and 1,129 cm^−1^, we assigned for CH_2_ bands from pure PEG [25]. The presence of residual PEG in porous silica matrix can be explained by not sufficient temperature for total removal of organic template. Recently, other groups showed that total elimination of PEG from the composites is available at the temperatures above 400°C [26,27]. Thermal stability of PEG-silica hybrid systems is influenced by a strong interaction between PEG and silica. Therefore, silica prevents the heat transfer to the inner parts of PEG [28]. For this reason, the vibrations of incorporated polymer chains are well expressed in Raman spectra. The peaks observed at 1,462 cm^−1^ correspond to C-H vibrations, while bands at 1,246 and 1,280 cm^−1^ are assigned to C-O-C bonds of PEG [25,29]. A broad band rising from 500 to 550 cm^−1^ represents mixed stretching and bending modes of Si-O-Si [30]. As seen in Figure 3b, a shoulder at 3,484 cm^−1^ corresponding to -OH vibrations of PEG is visible in Raman spectra of the film before its immersion in the silver nitrate solution. However, this band disappeared after the film was kept in AgNO_3_ solution for 17 h. This suggested that -OH groups of PEG were responsible for the reduction of silver ions to silver nanoparticles. Therefore, based on Raman measurement results, we hypothesized that residual PEG 400 could act as a reducing agent for silver cations and result in the formation of silver nanoparticles on the film surface. When such films were introduced into silver nitrate solution, the reduction of silver ions occurred through the oxidation of PEG 400 hydroxyl groups to aldehyde groups [20].

**Figure 4:**
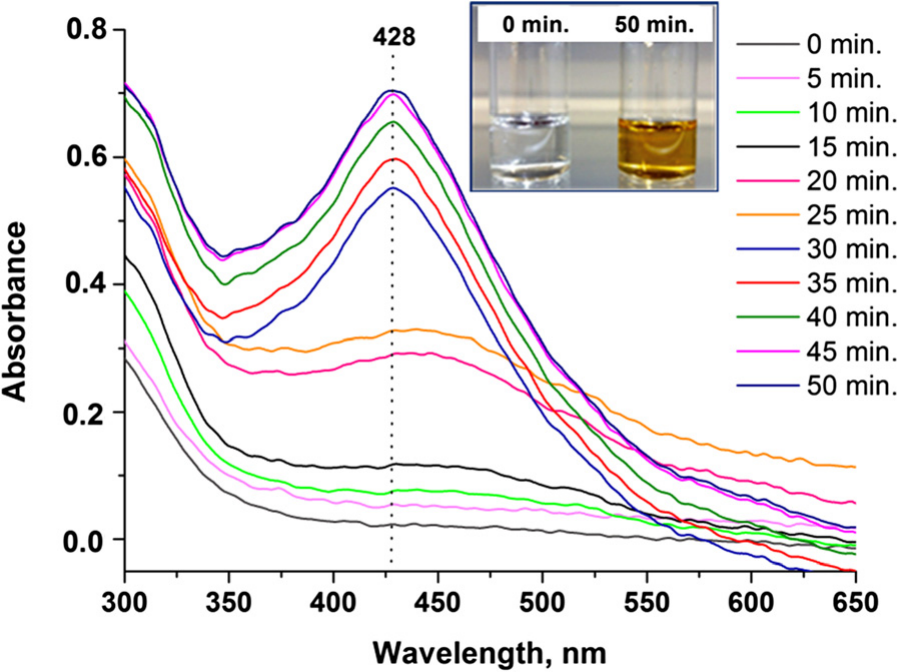
**Time-dependent UV-vis spectra showing the reduction of silver ions with PEG 400.**

To confirm that PEG 400 is able to act as a reducing agent, 1 M AgNO_3_ solution was mixed with pure PEG 400 (volume ratio 1:1). The reaction was monitored in real time by using UV-vis spectroscopy (Figure 4). At the beginning of the reaction, no absorption peak characteristic for silver nanoparticles was recorded. Nevertheless, a broad plasmon occurred in the spectra after 20 min from the moment when the silver precursor and the polymer were mixed. The plasmon increased with increasing the reaction time and was found to be located at 428 nm. The peak reached the maximum after 50 min and did not rise with longer reaction time, which indicated the end of the reaction. The colour of the solution changed from colourless to yellow which also suggested the successful synthesis of silver nanoparticles.

**Figure 5:**
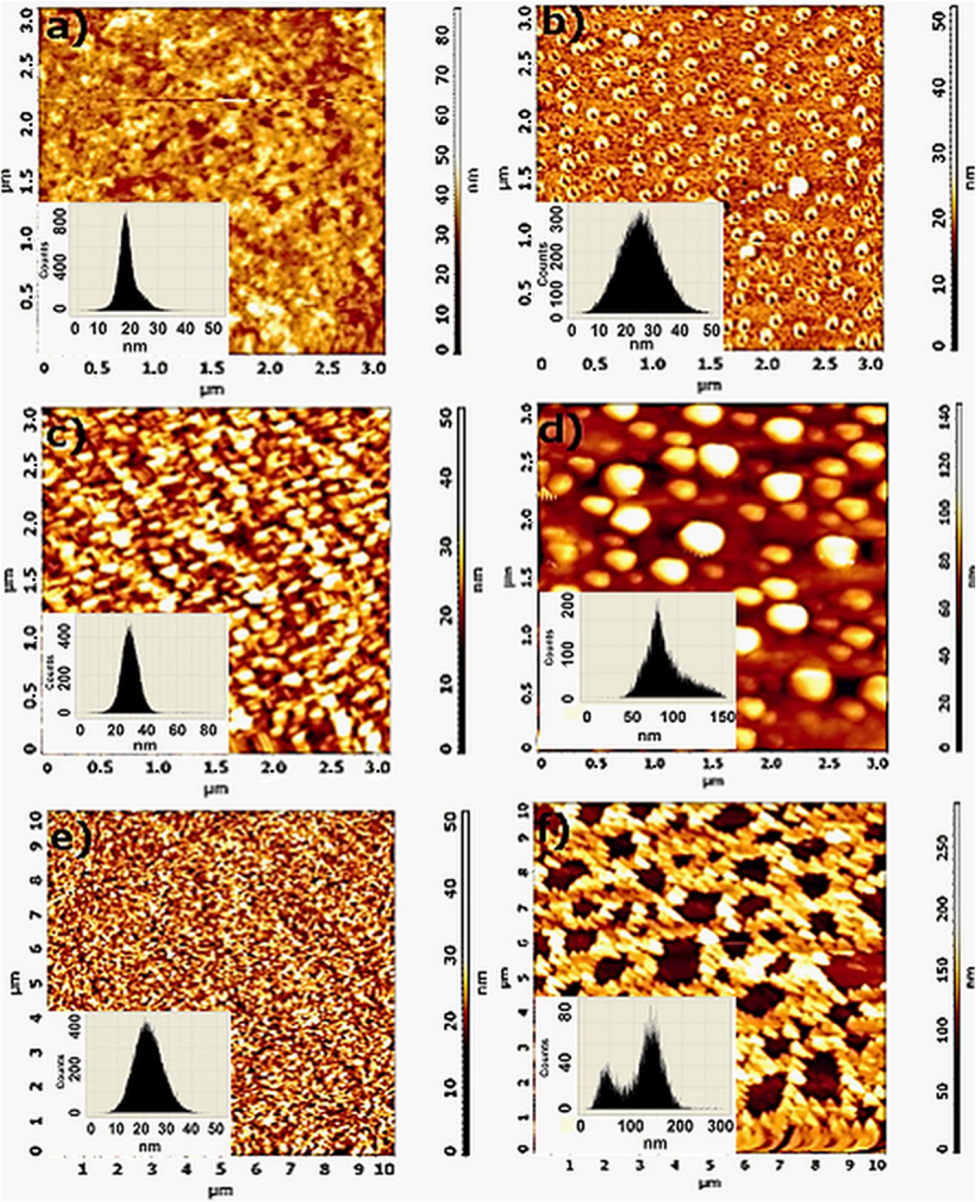
**Morphological characterization of hybrid silica films by AFM. (a)** A film prepared at a volume ratio of sol:PEG 1:0.05 after thermal treatment and **(b)** after exposure in silver nitrate solution for 17 h; **(c)** a film prepared at a volume ratio of sol:PEG 1:0.10 after thermal treatment and **(d)** after exposure in silver nitrate solution for 17 h; **(e)** a film prepared at a volume ratio of sol:PEG 1:0.15 after thermal treatment and **(f)** after exposure in silver nitrate solution for 17 h.

The proposed reduction of silver ions both in the solution and on the surface of the films can be explained by the following reaction:



Figure 5a,c,e shows the morphology of the silica films and the histograms of surface roughness. As it is seen from atomic force microscopy (AFM) measurements, the roughness of the silica films increases with increasing the concentration of PEG 400: they were equal to 18, 30 and 23 nm for the films prepared using lowest, middle and highest amount of PEG 400, respectively. Morphological characterization of the silver nanoparticle-decorated silica films revealed that the change of PEG 400 amount in the sol results in the different structures of AgNp formed on the film surfaces (Figure 5b,d,f). Silica films, prepared by using sol/PEG 400 ratio 1:0.05 (*v*:*v*), were decorated with nanosized silver rings, while the films prepared using sol/PEG 400 ratio 1:0.10 (*v*:*v*) were found to have spherical silver nanoparticles on the surface. The third type of the films sol/PEG 400 ratio 1:0.15 (*v*:*v*) was found to have the network of silver nanoparticles formed on the surface of the film. AFM height histograms showed the height distribution of silver nanoparticles of around 25, 70 and 150 nm for rings, spherical and self-assembled nanoparticle-decorated silica films, respectively.

**Figure 6:**
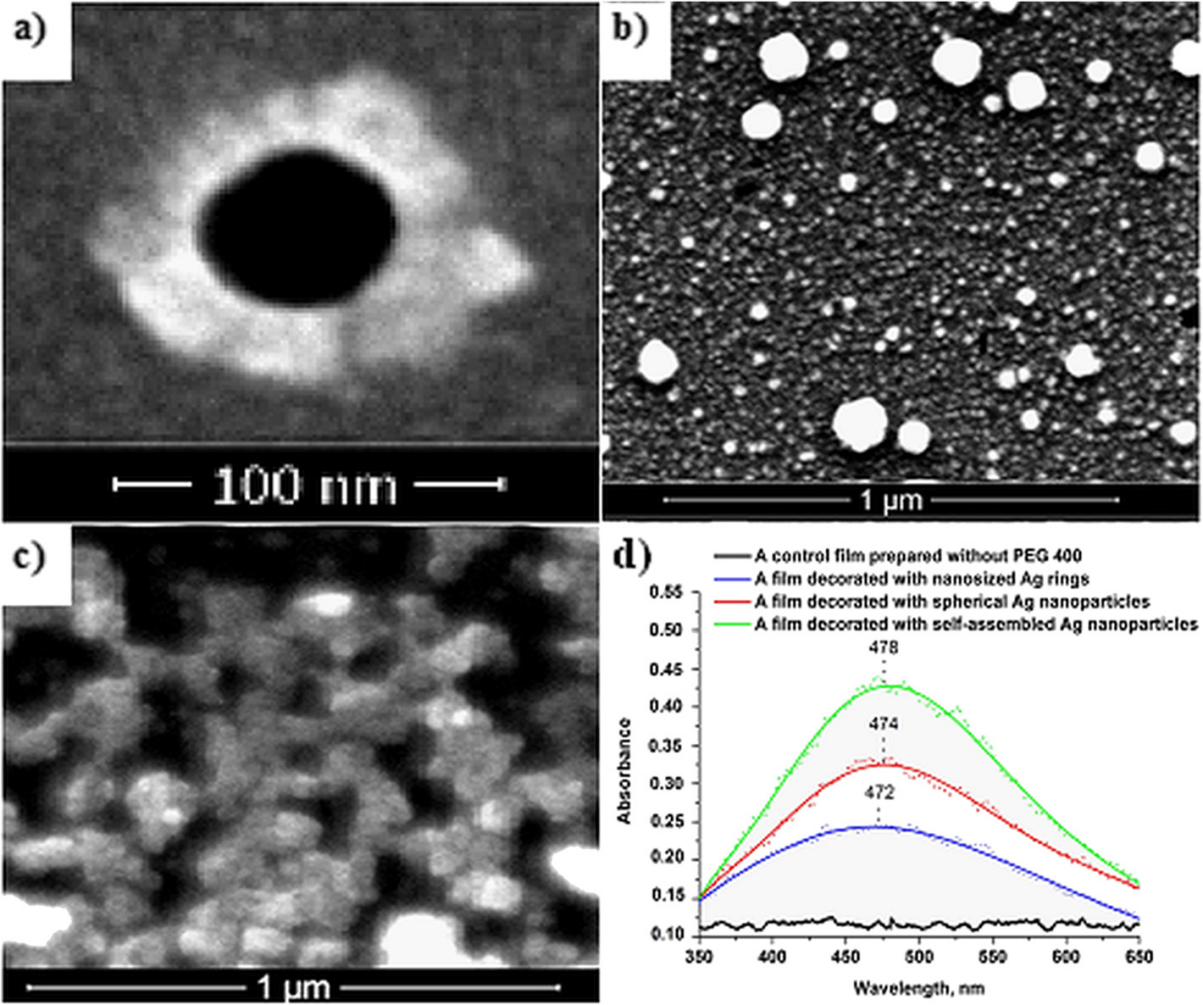
**SEM and UV-vis characterization of silver nanoparticle-decorated silica films. (a)** SEM image of nanosized silver ring-decorated silica film; **(b)** SEM image of spherical silver nanoparticle-decorated silica film; **(c)** SEM image of self-assembled silver nanoparticle-decorated silica film; **(d)** UV-vis spectra of silver nanoparticle-decorated silica films.

In all cases, the reduction of silver nanoparticles starts inside the pores, where the formation of primary nanoparticles takes place. Those particles are the nucleation centres and catalyze the reduction of other Ag cations around them. The lowest amount of organic template leads the growth of silver nanoparticles along the edges of the pores thus resulting in nanosized ring-like silver structures. When the amount of PEG 400 is increasing, reduction continues and results in spherical silver nanoparticle-filled pores. According to that high amount of polyethylene glycol template influencing the increase of interconnection between pores [27], a self-assembled structure of silver nanoparticles is formed on the surface of the third type of the film. The formation of nanosized silver rings, spherical and self-assembled silver nanoparticles on the film surfaces was also confirmed by the scanning electron microscopy (SEM) measurements (Figure 6a,b,c).

The optical characterization of all types of the films is presented in Figure 6d. Broad shoulders in UV-vis spectra at around 472 to 478 nm wavelength are typical for silver nanostructures. Recently, it has been reported that different nanostructures, such as silver spheres, rods, discs, triangles or truncated structures exhibit respectively mono-, di-, tri-, tetra- or multiple surface plasmon resonances in UV-vis absorption spectra, which result from individual dipole and/or quadrupole plasmon modes [31]. Due to the size and/or the shapes of the nanoparticles, the resonance conditions change and result in a shifted or broadened UV-vis spectra. Usually, the formation of AgNp is indicated by the reddish-brown colour [32] of solution, and in our case, all types of the films were found to be brownish. It is obvious from UV-vis measurements that absorption of hybrid films is increasing with increasing the amount of PEG 400. It is due to that after thermal treatment of the films, more residual PEG 400 stays entrapped into the pore channels and results in increasing density of silver nanoparticles on the film surface. To confirm that silver ions are reduced by PEG 400, control UV-vis spectra of PEG 400-free film were recorded. No plasmon in the range common for AgNp was observed, so this result led us to confirm the hypothesis that PEG 400 is responsible for the reduction of silver ions.

Silver nanoparticle-decorated silica films were tested as substrates for surface-enhanced Raman spectroscopy using 1% aqueous solution of crystal violet dye as a test material.

**Figure 7:**
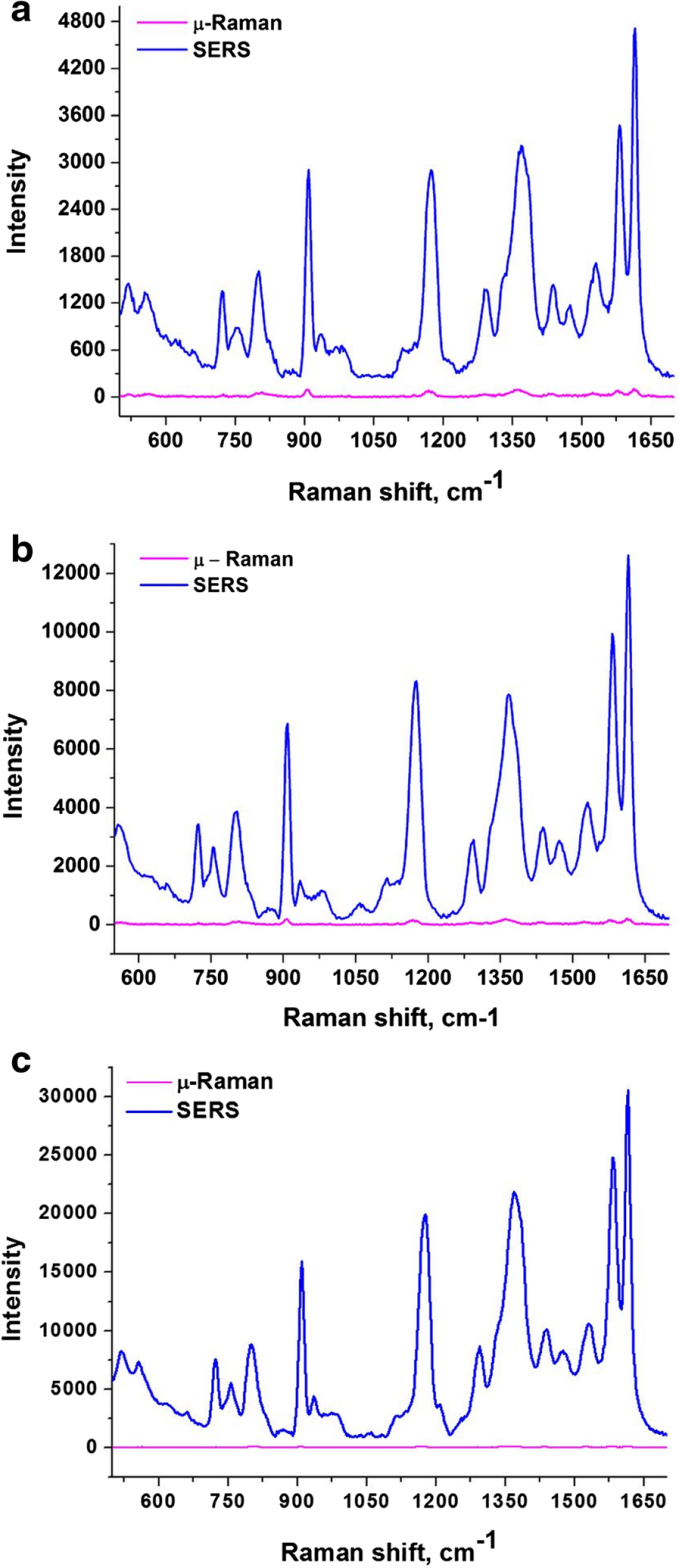
**Raman and SERS measurements of crystal violet solution adsorbed. (a)** Nanosized silver ring-decorated silica film; **(b)** spherical silver nanoparticle-decorated silica film; **(c)** self-assembled silver nanoparticle-decorated silica film.

It is seen from Figure 7 that the highest enhancement of test molecules (up to 200 times) was observed on the film, decorated with self-assembled silver nanoparticles. Highest amount of PEG 400 leads the formation of AgNps in a close proximity and result in a high content of dipole type plasmonic structures. The dipole-type plasmonic antennas produce high-intensity electromagnetic field and result in a high enhancement of Raman signal. Spherical silver nanoparticle-decorated substrate demonstrated the enhancement of around 100 times compared with micro-Raman, while nanosized silver ring-decorated films showed an enhancement of Raman signal of around 50 times. The low enhancement factor of nanosized silver ring-decorated substrates probably can be explained by relatively a large distance between the rings and as a result a low density of hot spots on the substrate in the area where the laser beam is focused.

It is important to note that the SERS and micro-Raman spectra we demonstrate in Figure 7 are an experimental spectra and we use the relation between spectra to estimate a SERS enhancement produced by different substrates. It is not a single-molecule enhancement spectra, because in order to determine an enhancement factor of single molecule, we must know the number of molecules participating in scattering cross-section, and this calculation is out of the scope of this work.

## Conclusions

A novel controllable synthesis of porous silica films decorated with silver nanoparticles using sol-gel technique with different amounts of PEG 400 was demonstrated. It was found that variation of PEG 400 amount in the sols allows the creation of different patterns of silver nanoparticles on the film surfaces. It was successfully synthesized silver nanorings, spherical and networked self-assembled structures of silver nanoparticles. Hybrid films were tested as SERS substrates, and measured relative Raman scattering enhancement was up to 200. Synthesized SERS substrates were used for protein SERS investigations and demonstrated a good reproducibility. We believe that further works in application of these novel nanostructured silver structures will open a new opportunity in the field of nanoplasmonics.
